# Real-World Impact of Initial Dual Bronchodilation on Exercise Physiological Response and Health-Related Quality of Life in Newly Diagnosed, Treatment-Naïve Chronic Obstructive Pulmonary Disease

**DOI:** 10.3390/medicina62030531

**Published:** 2026-03-12

**Authors:** Ieva Dimiene, Deimante Hoppenot, Airidas Rimkunas, Neringa Vaguliene, Kristina Bieksiene, Marius Zemaitis, Kestutis Malakauskas, Skaidrius Miliauskas

**Affiliations:** 1 Department of Pulmonology, Medical Academy, Lithuanian University of Health Sciences, 44307 Kaunas, Lithuania; deimante.hoppenot@lsmu.lt (D.H.); neringa.vaguliene@lsmu.lt (N.V.); kristina.bieksiene@lsmu.lt (K.B.); marius.zemaitis@lsmu.lt (M.Z.); kestutis.malakauskas@lsmu.lt (K.M.); skaidrius.miliauskas@lsmu.lt (S.M.); 2 Laboratory of Pulmonology, Department of Pulmonology, Medical Academy, Lithuanian University of Health Sciences, 44307 Kaunas, Lithuania; airidas.rimkunas@lsmu.lt

**Keywords:** treatment-naïve, COPD, CPET, SF-36, dual bronchodilation, beta-blockers

## Abstract

*Background and Objectives:* Dual bronchodilation in chronic obstructive pulmonary disease (COPD) has demonstrated beneficial effects on health-related quality of life (HRQoL) and exercise-related outcomes. Real-world evidence in treatment-naïve COPD remains limited. *Materials and Methods*: Forty-six COPD patients and 23 age-, gender-, BMI-, and cardiovascular comorbidity–matched controls underwent spirometry, plethysmography, symptom-limited incremental cardiopulmonary exercise testing (CPET), and the 36-item Short-Form Health Survey (SF-36). Following baseline assessment, COPD patients received tiotropium/olodaterol as part of routine practice. Thirty-two patients underwent repeated examinations at 12 weeks. Baseline differences between the COPD and control groups were assessed, and longitudinal changes in pulmonary function, CPET, and SF-36 were evaluated in COPD patients. *Results*: Compared with controls, COPD patients had lower peak oxygen uptake (VO_2_; 17.4 ± 4.4 vs. 22.8 ± 4.5 mL/kg/min, *p* < 0.001) and oxygen pulse (11.5 ± 3.5 vs. 14.0 ± 2.4 mL/beat, *p* = 0.003), failed to reach 80% of predicted values, and exhibited worse ventilatory efficiency (*p* < 0.001). SF-36 scores in the COPD group were lower across all domains. After 12 weeks of tiotropium/olodaterol, pulmonary function improved significantly. CPET was performed at comparable efforts at both visits. Peak VO_2_ increased from 70 ± 15 to 75 ± 16% predicted (*p* = 0.044), and peak oxygen pulse from 74 ± 16 to 79 ± 16% predicted (*p* = 0.015). VE/MVV decreased from 0.77 ± 0.23 to 0.69 ± 0.15 (*p* = 0.03). Higher baseline VE/MVV predicted a larger improvement after treatment (B = 0.71, *p* < 0.001), while beta-blocker use had no effect on the change of VE/MVV. SF-36 physical functioning and health change scores improved (both *p* < 0.01). *Conclusions*: At diagnosis, COPD was associated with impaired exercise physiology and reduced HRQoL. Dual bronchodilation improved exercise responses and perceived physical functioning. Beta-blocker use was not associated with changes in breathing reserve, supporting the use of cardioselective agents when indicated.

## 1. Introduction

Chronic obstructive pulmonary disease (COPD) is one of the leading causes of morbidity and mortality globally and is associated not only with progressive airflow limitation but also with deterioration in exercise capacity and health-related quality of life (HRQoL) [1,2,3,4]. Recent proposals highlight the importance of identifying the development of this disease even before clear airflow limitation is present and highlight the need to consider causal factors beyond cigarette smoking. Thus, COPD is increasingly understood as a heterogeneous condition with multiple pathogenic mechanisms and clinical presentations, which may require different preventive and therapeutic strategies [5]. Moreover, failing lung regeneration has been suggested as an important contributing mechanism to progressive functional decline in COPD [6]. Therefore, early detection and appropriate management of COPD are essential for improving outcomes.

In patients with COPD, exercise limitation is multifactorial and results from dynamic lung hyperinflation, impaired synergy between respiratory and peripheral muscles related to limited energy supply, lower-limb muscle dysfunction, and the presence of cardiovascular comorbidities [7,8,9]. Cardiopulmonary exercise testing (CPET) provides insights into the mechanisms of dyspnea in COPD and might help identify secondary or coexistent non-respiratory causes (e.g., cardiovascular comorbidities, myopathy, or deconditioning) that contribute to exercise limitation [10]. Moreover, reduced exercise capacity is a well-established predictor of increased mortality in COPD [11].

Clinical trials have demonstrated that treatment with combinations of long-acting muscarinic antagonists (LAMAs) and long-acting β_2_-agonists (LABAs) improves exercise capacity and physical activity in COPD [12]. Most studies have assessed the effects of LAMA/LABA therapy on exercise endurance time and walking distance [13,14,15,16]; however, not many have reported detailed CPET-derived physiological responses [17,18]. Given the high prevalence of cardiovascular comorbidities in COPD, evidence regarding whether concomitant beta-blocker therapy modifies CPET-derived physiological responses to dual bronchodilation remains limited. Although a subset of recent studies suggests that cardioselective beta-blockers are generally safe and do not exhibit clinically relevant adverse interactions with bronchodilator therapy, some reports continue to raise concerns about potential negative effects, contributing to their ongoing underuse in COPD [19,20,21].

Together with exercise-based outcomes, a comprehensive assessment of patient-reported health status and functioning is essential to fully capture the systemic impact of COPD and its comorbidities [22]. The SF-36 is a valid instrument for evaluating HRQoL in COPD patients [23]. Studies have shown that COPD patients have worse SF-36 scores than the general population and patients with other chronic conditions [4]. Even though clinical trials involving COPD medications have focused mostly on disease-specific measures, SF-36 has also proven useful for assessing treatment efficacy [24]. However, data supporting its use for evaluating the effects of specific medications on HRQoL remain limited.

Although COPD significantly affects exercise physiology and HRQoL, and dual bronchodilation has been shown to improve these outcomes, current evidence is mostly derived from trials including previously treated patients. The lack of real-world data on treatment-naïve patients represents an important gap in the literature and provides the rationale for our study. Moreover, as COPD is a multifactorial systemic disease, evaluating its impact beyond ventilatory impairment and disease-specific patient-reported outcomes is essential.

The primary endpoints of this study were (1) changes in physiological responses at peak exercise during symptom-limited incremental CPET and (2) changes in HRQoL after 12 weeks of tiotropium/olodaterol (TIO/OLO) treatment in patients with COPD. Secondary endpoints included (1) evaluation of the impact of beta-blocker use on ventilatory responses to TIO/OLO and comparison of (2) exercise physiological responses and (3) HRQoL between patients with COPD and age-, sex-, body mass index (BMI)-, and cardiovascular disease–matched non-COPD controls.

## 2. Materials and Methods

### 2.1. Study Design and Participants

This was a prospective, real-world observational study conducted at the Hospital of Lithuanian University of Health Sciences Kauno Klinikos, in the Department of Pulmonology. We enrolled 46 newly diagnosed treatment-naïve patients with moderate-to-severe COPD (study group) and 23 non-COPD individuals of similar age, sex and cardiovascular comorbidities (control group). The inclusion and exclusion criteria for COPD patients have been described previously [25]. The control group consisted of subjects without respiratory diseases. Control subjects matched to the COPD group for age, sex, BMI, and stable cardiovascular comorbidities were included.

Patients with COPD underwent pulmonary function testing (spirometry and body plethysmography), completed the SF-36, and performed symptom-limited incremental CPET before and after 12 weeks of treatment with a fixed-dose combination (FDC) of TIO/OLO 5/5 µg once daily. Changes in the results of these tests were evaluated. Importantly, only 32 subjects in the COPD group underwent the investigation after 12 weeks of dual bronchodilation (completers). Other patients were lost to follow-up (unable to be contacted to plan the follow-up visit), refused to repeat the tests due to personal circumstances, did not adhere to the treatment regimen or needed to be referred for a more detailed, timely assessment or treatment correction of existing stable cardiovascular comorbidities (non-completers). Baseline characteristics were compared between completers (*n* = 32) and non-completers (*n* = 14) to evaluate potential attrition bias. The subjects in the control group performed spirometry, body plethysmography, completed the SF-36 and performed CPET once. We compared the characteristics and results of the SF-36 and CPET between the study (at baseline) and control groups.

Clinical Trial Registration: The study was registered in the National Institutes of Health trial registry of the United States https://clinicaltrials.gov/. The identification number is NCT06072690. Registration date: 10 October 2023.

### 2.2. Pulmonary Function Testing

We measured the forced expiratory volume in 1 s (FEV_1_), forced vital capacity (FVC) and the FEV_1_-to-FVC ratio with a Ganshorn spirometry device (Ganshorn Medizin Electronic, Niederlauer, Germany). Body plethysmography was performed in a Ganshorn Power Cube Body + plethysmography chamber (Ganshorn Medizin Electronic, Niederlauer, Germany). For body plethysmography, we assessed the following parameters: individual values in L or % (depending on the measured parameter) and % of predicted values (% pred.) of residual volume (RV), total lung capacity (TLC), RV/TLC, functional residual capacity (FRC) and FRC/TLC.

### 2.3. Cardiopulmonary Exercise Testing

We performed a symptom-limited, arterial blood pressure, pulse oximetry and electrocardiogram-monitored incremental CPET using a cycle ergometer (Schiller CS-200, Munich, Germany). The work rate (WR) increase (W/min) was selected based on physical activity in everyday life (the same WR increase was applied during the CPET before the treatment and after 12 weeks of treatment for COPD patients). Following the resting and unloaded phases, all patients proceeded to the loaded phase and then pedaled with equal encouragement until symptom limitation (e.g., dyspnea or inability to maintain a speed of more than 50 revolutions per minute) or the appearance of test termination criteria (e.g., blood pressure > 240 mmHg or repetitive ventricular extrasystoles). All patients completed the test in the unloaded phase. At rest, we evaluated heart rate (HR; beats per minute [bpm]), oxygen saturation with pulse oximetry (SpO_2_), BORG-10 scores for dyspnea and scores for leg pain. The following parameters at peak exercise were assessed: SpO_2_, BORG-10 scores for dyspnea and scores for leg pain, HR (beats per minute (bpm) and % pred.), WR (W), oxygen uptake (VO_2_) (mL/kg/min, L/min and % pred.), oxygen (O_2_) pulse (mL/beat and % pred.), the slope of VO_2_ relative to the increase in WR (ΔVO_2_/ΔWR), minute ventilation (VE) (L/min and % pred.), VE-carbon dioxide output slope (VE/VCO_2_), VE-to minute voluntary ventilation ratio (VE/MVV) and exercise endurance time (EET, s). MVV was calculated as FEV_1_ × 35, where FEV_1_ was measured before the CPET. Due to technical restrictions, we did not perform arterial blood gas (ABG) tests and inspiratory capacity (IC) maneuvers for our subjects. As not all patients with COPD have achieved an anaerobic threshold (AT) due to early fatigue, dyspnea or test termination criteria, we did not evaluate the parameters at AT to prevent measurement inaccuracies. While maximality criteria were not consistently achieved, effort at baseline and follow-up was comparable, enabling assessment of within-subject changes in peak responses and exercise physiology.

### 2.4. Health-Related Quality of Life

The Lithuanian-validated version of the SF-36 was used to evaluate HRQoL [26]. The questionnaire covers eight main domains: physical functioning, role limitations due to physical health problems, role limitations due to personal or emotional problems, bodily pain, emotional well-being, social functioning, energy (fatigue), and general health. As the ninth domain, health change, has also been used in previous studies to assess changes in health status compared with one year prior, we also included it in our assessment [23]. The scores in all domains range from 0 to 100, with higher scores indicating better results.

### 2.5. Statistical Analysis

For statistical analysis, we used IBM SPSS Statistics for Windows, version 30.0.0.0 (IBM Corp., Armonk, NY, USA). To compare numerical variables between different groups (study vs. control and COPD patients who attended the follow-up visit vs. COPD patients who did not attend the follow-up visit), we used the independent t-test for normally distributed variables and the Mann-Whitney test for non-normally distributed variables. To compare categorical variables between groups, Pearson’s chi-square test was used when expected cell counts were ≥5, while Fisher’s exact test was applied when chi-square assumptions were violated. Differences in reasons for CPET termination between COPD and non-COPD participants were evaluated using Fisher’s exact test due to small cell counts. To evaluate changes in the measured parameters from baseline to 12 weeks of treatment with dual bronchodilation in COPD patients, we used the paired samples t-test for normally distributed data and the Wilcoxon signed-rank test for non-normally distributed data. Numerical values were presented as the means ± standard deviations (SDs) or medians with interquartile ranges (IQRs). Multivariable linear regression was used to evaluate changes in VE/MVV, with baseline VE/MVV and beta-blocker use included as covariates. The results were considered statistically significant at *p* < 0.05.

## 3. Results

### 3.1. Characteristics of COPD Patients and Non-COPD Individuals

We compared 46 newly diagnosed treatment-naïve patients with moderate-to-severe COPD with 23 non-COPD subjects. Age, sex and BMI did not differ significantly between groups (*p* = 0.263, *p* = 0.167 and *p* = 0.18, respectively). Both groups had similar cardiovascular comorbidity profiles, with arterial hypertension and dyslipidemia being the predominant underlying conditions. A comparable number of subjects were on beta-blockers in both groups; all patients and control group subjects used cardioselective beta-blockers. The characteristics of participants are presented in Table 1.

### 3.2. Comparison of Completers and Non-Completers

A significant part (~30%) of COPD patients did not attend the 12-week follow-up visit (reasons mentioned in the Section 2). Baseline characteristics were compared between COPD patients who attended the 12-week follow-up visit (completers; *n* = 32) and those who did not (non-completers; *n* = 14) to assess potential attrition bias. There were no significant differences between groups with respect to age (59.8 ± 8.3 vs. 63.8 ± 6.8 years), BMI (27.2 ± 5.2 vs. 27.7 ± 3.7 kg/m^2^), sex distribution, COPD severity, comorbidities, or beta-blocker use (all *p* > 0.05). The key baseline exercise parameters were also comparable, including peak VO_2_% pred. (70.3 ± 14.7 vs. 69.6 ± 20.7) and peak VE/MVV (0.68 ± 0.2 vs. 0.67 ± 0.2; both *p* > 0.05).

### 3.3. Cardiopulmonary Exercise Testing: COPD Patients Versus Non-COPD Individuals

Significant differences between groups were observed in the majority of CPET parameters. At peak exercise, the COPD group reached lower O_2_ pulse, VO_2_ (both across all measurements), higher VE/MVV and VE/VCO_2_ slope. Patients with COPD achieved a lower VE (L/min) at peak exercise than non-COPD subjects did. Individuals with COPD more frequently terminated exercise due to dyspnea, while controls—due to peripheral fatigue or cardiac reasons (*p* < 0.001). The detailed results are presented in Table 2.

### 3.4. Health-Related Quality of Life: COPD Patients Versus Non-COPD Individuals

Subjects in the COPD group had significantly lower SF-36 scores across all domains compared with those in the non-COPD group. The largest differences between groups were observed in physical functioning, role limitations due to physical health and role limitations due to emotional problems (all *p* < 0.001). The detailed results are presented in Table 3.

### 3.5. Pulmonary Function Tests: Changes After 12 Weeks of Dual Bronchodilation

**Figure 1:**
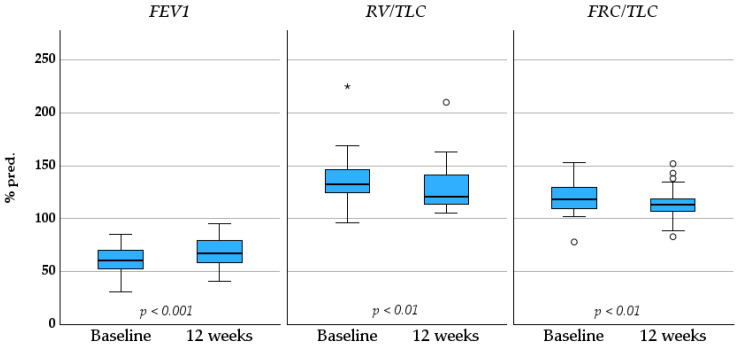
Baseline-to-12-week changes in pulmonary function measurements in COPD patients. * *p*-value < 0.05 indicates statistically significant results. The values in the table are presented as the means ± SDs or medians (IQRs), as appropriate. Abbreviations: FEV_1_—forced expiratory volume in 1 s, RV—residual volume, TLC—total lung capacity, FRC—functional residual capacity, % pred.—percentage of predicted value.

After 12 weeks of treatment, significant improvement in pulmonary functional parameters was observed in patients who attended the follow-up visit (*n* = 32). In spirometry, FEV_1_ increased from 2.08 ± 0.61 to 2.33 ± 0.68 L (*p* < 0.001) and from 61 ± 14 to 69 ± 15% pred. (*p* < 0.001). FVC also increased from 3.50 ± 0.91 to 3.62 ± 0.89 L (*p* < 0.04) and from 80 ± 14 to 83 ± 15% pred. (*p* = 0.014). While RV and FRC did not reduce significantly, there was a trend towards significance (both *p* < 0.07). Furthermore, significant decreases in RV/TLC from 133 (IQR 124–147) to 121 (IQR 114–142) % pred. and in FRC/TLC from 120 ± 15 to 114 ± 15% pred. (both *p* < 0.01) were observed. TLC remained stable (from 107 ± 20 to 108 ± 14% pred., *p* = 0.629). The most pronounced changes in pulmonary function measurements are presented as median values in the boxplot (Figure 1).

### 3.6. Cardiopulmonary Exercise Testing: Changes After 12 Weeks of Dual Bronchodilation

During both visits, symptom-limited incremental CPET was performed at comparable efforts—RER, HR, BORG and leg pain scores at peak exercise did not differ significantly, peak WR was comparable between tests, with identical medians and similar interquartile ranges (120 [90–140] vs. 120 [100–139] W; *p* = 0.05). We found the improvement in several cardiopulmonary parameters at peak exercise after 12 weeks of treatment. Significant changes were observed in VO_2_ (both absolute value and % pred.), with a trend toward significance noted for VO_2_ per kilogram (VO_2_/kg; *p* = 0.058). An improvement in O_2_ pulse was observed in both absolute and % pred. values (*p* < 0.05). From ventilatory measurements, the mean value of VE/MVV decreased (*p* = 0.03), improving breathing reserve. A detailed summary of the results is provided in Table 4.

### 3.7. Baseline Predictors of Ventilatory Response to Dual Bronchodilation

In multivariable linear regression analysis including baseline VE/MVV and beta-blocker use as covariates, only baseline VE/MVV was independently associated with change in VE/MVV, while beta-blocker use showed no significant association. The model explained 54% of the variance in VE/MVV change (Table 5).

### 3.8. Health-Related Quality of Life: Changes After 12 Weeks of Dual Bronchodilation

After 12 weeks of dual bronchodilation, physical functioning and health change scores increased significantly in patients with COPD (both *p* < 0.01). The other seven domains of the SF-36 did not differ. A comprehensive summary of the results is provided in Table 6.

## 4. Discussion

### 4.1. Differences in Exercise Physiological Responses and Health-Related Quality of Life: COPD Versus Non-COPD Individuals

In our study, a substantial part of CPET responses demonstrated significant differences between treatment-naïve patients with COPD at the time of diagnosis and non-COPD subjects of comparable age, sex, BMI and cardiovascular comorbidities. Patients with COPD not only exhibited worse ventilatory measurements but also showed impaired cardiovascular parameters at peak exercise and achieved lower WRs (Table 2).

Previously published data indicated that differences in CPET responses between individuals with and without COPD vary depending on disease severity and the characteristics of the control group. In the study by Soumagne et al., patients with mild COPD (*n* = 20) demonstrated lower peak WR and peak V̇O_2_ during symptom-limited incremental CPET compared with controls of similar age, sex, and comorbidities (*p* < 0.05). However, HR, O_2_ pulse, VE, and VE/V̇CO_2_ at peak exercise did not differ significantly between groups [27]. In cohorts with predominantly moderate-to-severe COPD, more differences in CPET-derived measurements have been reported. Cortopassi et al. observed lower peak V̇O_2_ (1.10 ± 0.38 L/min vs. 1.72 ± 0.47 L/min), O_2_ pulse (10.6 ± 3.7 vs. 14.3 ± 2.7 mL/beat), HR (113 ± 15 vs. 148 ± 18 bpm), and WR (79.6 ± 36.9 vs. 143.2 ± 31.7 W, all *p* < 0.01) in patients with COPD (*n* = 18) compared with healthy age-matched controls (*n* = 15). Although inspiratory capacity maneuvers were performed during CPET in that study, other ventilatory parameters were not compared between groups. Notably, they also reported a markedly shorter CPET duration in patients with COPD, whereas no such difference was observed in our study [28]. This discrepancy may be explained by differences in control group recruitment. While Cortopassi et al. enrolled healthy volunteers recruited through hospital advertisements, our control group consisted of individuals with cardiovascular comorbidities like those of the COPD group, referred by general practitioners. This approach may have reduced differences in exercise duration, allowing for a more clinically relevant comparison. In the study by Barron et al., patients with COPD (*n* = 25) demonstrated a lower mean (±standard error) peak V̇O_2_ compared with healthy controls (*n* = 134) (1.356 ± 0.073 L/min vs. 1.937 ± 0.031 L/min, *p* < 0.05). A statistically significant difference in peak O_2_ pulse was also observed between the groups (10.7 ± 0.5 mL/beat in COPD patients vs. 13.8 ± 0.2 mL/beat in controls). Similarly to our findings, VE/VCO_2_ slope in this study was higher in the COPD group than in healthy controls (33.6 ± 1.1 vs. 26.5 ± 0.5, *p* < 0.05). Although differences in the V̇O_2_/WR slope were evaluated, no statistically significant between-group differences were detected, in contrast to our findings [29]. This may be related to differences in study design, including group sizes and control group selection.

To summarize the findings of previous studies and this study, the impairment in CPET responses progressively increases with COPD severity, indicating that both ventilatory and cardiovascular components are critical exercise-limiting factors. By comparing newly diagnosed treatment-naïve COPD patients with non-COPD individuals matched with age, sex, BMI, and cardiovascular comorbidities, our findings suggest that at the time of diagnosis, COPD is associated with altered cardiocirculatory responses during exercise, beyond the presence of cardiovascular comorbidities alone.

We observed significant differences in HRQoL between treatment-naïve moderate-to-severe COPD patients and non-COPD individuals matched with age, sex, BMI and cardiovascular comorbidities across all domains of SF-36 (Table 3).

Similarly to our results, Bentsen et al. reported that COPD patients waiting to begin a pulmonary rehabilitation program had lower scores across all SF-36 domains than the general population (*p* < 0.001). The studied subjects were of similar age and sex; however, unlike our study, it was not specified whether the COPD patients and healthy individuals had similar cardiovascular comorbidities [4]. While previous studies have rarely focused on newly diagnosed treatment-naïve patients, a multicenter Canadian study evaluated the SF-36 in newly diagnosed (previously undiagnosed) COPD patients and healthy controls and revealed that the COPD group had a significantly poorer global health status (the unadjusted mean difference in the total score was −19.7, *p* < 0.001). However, the methodology for scoring SF-36 in this study differed from standard approaches, and a comprehensive domain-level analysis was not provided [30]. The findings of the latter study and our cohort highlight the substantial impairment in HRQoL that patients with COPD already experience by the time they seek medical advice or receive their diagnosis.

### 4.2. The Impact of Dual Bronchodilation on Lung Function, Exercise Physiological Responses and Health-Related Quality of Life in COPD Patients

The positive effect of dual bronchodilation on lung function, as measured by spirometry and body plethysmography, has been demonstrated in previous studies, including those evaluating TIO/OLO [31,32,33,34]. In our study, the most significant improvements after 12 weeks of treatment with TIO/OLO were observed in FEV_1_, RV/TLC, and FRC/TLC (Figure 1), which is consistent with the previous findings and aligns with the expected effects of dual bronchodilation therapy.

We observed significant improvements in a subset of cardiopulmonary parameters during the symptom-limited incremental CPET at peak exercise after 12 weeks of treatment (Table 4). In our study, the mean improvement in peak VO_2_ (% pred.) was 5 ± 13% (*p* = 0.044), whereas the O_2_ pulse (% pred.) increased by 5 ± 12% (*p* = 0.015). While the peak HR remained unchanged, this suggests an improvement in stroke volume and overall cardiovascular efficiency rather than chronotropic compensation alone. This may indicate enhanced cardiac function during exertion, likely due to reduced ventilatory limitation, as peak VE/MVV has decreased by 0.083 ± 0.21 (*p* = 0.03). However, we did not perform IC maneuvers, which could have provided valuable information about dynamic hyperinflation and its impact on O_2_ pulse and VO_2_ at peak exercise.

We found that in one COPD study, a change in VO_2_ at maximal exercise of approximately 40 mL/min (corresponding to a 4 W change in work rate) was considered a minimal clinically important difference (MCID) [35]. In our study, the mean change in peak VO_2_ was 107 ± 256 mL/min, suggesting a potentially clinically meaningful improvement. However, the relatively large variability in individual results indicates heterogeneity in treatment response. While other CPET-derived parameters (e.g., O_2_ pulse and VE/MVV) do not have well-established MCIDs, the direction of these physiological changes observed in our study suggests a favorable response to treatment. Nevertheless, their clinical significance should be interpreted cautiously.

We found several studies in which a comprehensive analysis of CPET parameters was performed to evaluate the effect of dual bronchodilation in COPD patients. In the placebo-controlled crossover trial by Stringer et al., after two weeks of treatment with glycopyrrolate/formoterol fumarate, COPD patients (*n* = 48) across all GOLD stages had longer EET, greater IC at all time points during CPET, and greater tidal volume (VT) and VE at isotime than did placebo patients (*p* < 0.05). However, no significant differences in cardiovascular parameters (e.g., O_2_ pulse and VO_2_) at peak exercise were detected between the groups [17]. Cao et al. conducted a placebo-controlled crossover study in which patients with mild-to-severe COPD (*n* = 14) received TIO/OLO for one week and were compared to a placebo group. They did not find significant differences in CPET measurements—either cardiac or ventilatory—between the groups at isotime [18]. Berton et al. analyzed the effect of TIO/OLO on CPET variables in patients with moderate-to-severe COPD (*n* = 20) in a placebo-controlled crossover study. CPETs were performed across three consecutive visits, each at least 48 h apart. Treatment with TIO/OLO resulted in significantly greater IC, lower end-expiratory and end-inspiratory lung volumes, and reduced VE/MVV at isotime, whereas the change in cardiovascular measurements did not differ significantly between the TIO/OLO and placebo groups (*p* > 0.05) [34]. However, the study designs, treatment durations, inclusion criteria and CPET protocols of the previously mentioned trials differed markedly from those of our real-world study, which likely contributed to the differences in findings. It is important to note that, although these trials compared pre- and post-treatment changes in constant-load exercise tests, peak-exercise measurements obtained during symptom-limited incremental CPET are also commonly used in clinical COPD research to evaluate treatment effects [36,37].

In this study, we also performed a multivariable linear regression to evaluate the effect of beta-blocker use on the change in VE/MVV after 12 weeks of dual bronchodilation, adjusting for baseline VE/MVV (Table 5).

The use of beta-blockers in our study did not have a significant impact on VE/MVV change, suggesting no measurable effect on ventilatory limitation. In our cohort, patients were treated with various cardioselective beta-blockers (e.g., metoprolol, bisoprolol, betaxolol, nebivolol) at different doses. Beta-blocker therapy was analyzed according to its presence or absence, and no dose-specific analyses were performed. Therefore, potential differences related to specific agents or dosing regimens cannot be entirely excluded. Earlier published results of trial by Mainguy et al. (*n* = 27) showed that the use of cardioselective beta-blocker bisoprolol was associated with modest worsening of dynamic hyperinflation in patients with moderate-to-severe COPD [38], a more recent randomized crossover study by Anderson et al. (*n* = 11) concluded that dynamic hyperinflation was not different between bisoprolol or celiprolol (after 4 weeks of treatment) or versus baseline in moderate-to-severe COPD subjects, also suggesting that treatment regimens including LAMA prevent bronchoconstriction when beta-blockade is used in COPD [39]. While we did not directly assess parameters of dynamic hyperinflation, the finding that the change in peak VE/MVV was independent of beta-blocker use supports the growing evidence that beta-blockers do not meaningfully impair ventilatory mechanics in COPD, particularly in patients receiving maintenance dual bronchodilation [40].

We observed that higher baseline VE/MVV predicted greater improvement in VE/MVV values after 12 weeks of treatment. This finding is consistent with previous studies demonstrating that patients with greater baseline ventilatory impairment have more marked functional limitation and may therefore experience larger physiological benefits when lung volumes are improved with bronchodilator therapy [41,42].

In a recent COPD and asthma study, an MCID of approximately 8–10 points for SF-36 domain scores was used, based on previous studies [30]. In our cohort, the SF-36 physical functioning and health change scores in COPD patients improved significantly during the 12-week treatment with TIO/OLO (Δ9 ± 15 points and Δ25 [IQR: 0–50] points, respectively; both *p* < 0.01). Therefore, the observed improvement in physical functioning meets the aforementioned MCID threshold, while the magnitude of change in the health change domain suggests a substantial perceived benefit. The improvement in physical functioning may reflect improved physiological responses in CPET resulting from enhanced bronchodilation during TIO/OLO therapy. Nevertheless, the responsiveness of specific SF-36 domains in COPD populations may vary, and results should be interpreted within this context. It is important to note that other SF-36 domains remained stable at follow-up, suggesting that 12 weeks may be insufficient to impact broader emotional or social well-being. Additionally, as a generic health-related quality-of-life instrument, the SF-36 may be less sensitive to disease-specific changes in COPD compared with respiratory-specific questionnaires.

However, the use of generic questionnaires in COPD populations may still be beneficial, particularly as increasing attention is being given to the extrapulmonary effects of COPD therapies [1]. We did not find studies where the SF-36 tool was used to evaluate the effect of specific dual bronchodilation therapies in COPD patients. However, there were two studies of large cohorts in which the Physical Functioning Questionnaire (PF-10), a validated subdomain of the SF-36 created to examine perceived limitations in physical functioning, was evaluated after 4–6 weeks of treatment with TIO/OLO. In both studies, physical functioning increased significantly—the mean changes from baseline were 10.2 and 16.6 points (both *p* < 0.05, respectively) [43,44]. In an earlier published study by Sato et al., the scores of all eight SF-36 domains, except for bodily pain, significantly improved during the first 3 or 6 months of treatment in newly diagnosed treatment-naïve COPD patients (*n* = 100). After 3 months, improvements in physical functioning, role limitations due to physical health, vitality, social functioning, and mental and general health scores were observed in comparison with those at baseline (*p* < 0.05) [24]. While the data from the latter study revealed improvements in more domains of the SF-36 after 3 months of COPD treatment than our study did, we assume that the disparities between the results of our cohort and those of Sato et al. might be due to differences in sample size, treatment options, and inclusion criteria (e.g., lower mean FEV_1_ in the Sato et al. cohort).

Summarizing the data from our study and previously published results, generic tools might provide useful information when assessing the impact of COPD treatment on HRQoL. Since physical functioning appears to be the domain that improves the most with treatment, shorter and easier-to-administer questionnaires such as the PF-10 may also be preferable.

It is important to mention that variations in concomitant medications and the severity or type of comorbidities may have influenced both baseline comparison of COPD and non-COPD groups and post-treatment outcomes in the COPD group. Such factors could act as potential confounders, affecting exercise performance and HRQoL responses independently of COPD treatment with dual bronchodilation. Therefore, the results should be interpreted considering these possible influences.

## 5. Limitations and Strengths of the Study

A major strength and novel aspect of this study is its real-world design, including only treatment-naïve patients with COPD at the time of diagnosis. Studies evaluating newly diagnosed COPD populations remain limited, and this approach allowed us to characterize early treatment responses in a clinically relevant cohort.

This study has several limitations that should be noted. First, the sample size was relatively small; however, we included only treatment-naïve patients with moderate-to-severe COPD, which has limited our ability to recruit more participants. The second limitation relates to the structure of the control group. Only age-, sex-, BMI-, and cardiovascular comorbidity-matched individuals without respiratory diseases were included for baseline comparisons. A parallel COPD group receiving alternative treatment was not included. Therefore, the study does not allow for direct comparative evaluation of different therapeutic strategies. Another important limitation is that a significant part of COPD patients did not attend a 12-week follow-up visit. This is a common limitation in real-world studies and may reflect challenges in early patient engagement or the need for concurrent assessment and treatment of comorbidities. To assess the potential impact of attrition bias, baseline characteristics of completers and non-completers were compared, and no statistically significant differences were observed between groups. Nevertheless, the possibility of unmeasured differences cannot be entirely excluded. From a physiological perspective, IC maneuvers were not performed during CPET due to technical constraints. As a result, dynamic hyperinflation could not be directly quantified, despite its recognized role as a major determinant of exertional dyspnea in COPD. Instead, ventilatory limitation was evaluated from surrogate parameters. In addition, ABG analysis was not performed. Therefore, changes in O_2_ and CO_2_ levels during exercise were not directly assessed. The absence of ABG data limits a more comprehensive evaluation of gas exchange abnormalities and the ability to distinguish between ventilatory and gas exchange contributions to exercise intolerance.

## 6. Conclusions

At the time of diagnosis, COPD was associated with impaired exercise physiology and reduced HRQoL. In this real-world setting, initiation of dual bronchodilation was associated with improvements in exercise responses and perceived physical functioning. Individuals with greater baseline ventilatory limitation tended to show larger improvements in breathing reserve during exercise. Concomitant beta-blocker therapy was not associated with changes in breathing reserve. Overall, these findings may indicate functional benefits of early dual bronchodilation in routine clinical care and are consistent with the use of cardioselective beta-blockers when indicated.

## Figures and Tables

**Table 1:** Demographic characteristics and concomitant diseases of COPD patients and non-COPD individuals.

Characteristic	COPD (*n* = 46)	Non-COPD (*n* = 23)	*p*-Value *
**Demographics**			
Age, years	61 ± 7.99	58.8 ± 8.08	0.283
Sex (male:female)	37:9	15:8	0.167
BMI, kg/m^2^	26.8 (24.28–30)	27.9 (25–32.5)	0.18
Smoking history, pack-years	30 (15–47.75)	0 (0–16)	<0.001
**Lung function**			
FEV_1_ (L)	1.96 ± 0.59	3.43 ± 0.77	<0.001
FEV_1_ (% pred.)	59 ± 14	103 ± 12	<0.001
FVC (L)	3.37 ± 0.88	4.31 ± 0.92	<0.001
FVC (% pred.)	78 ± 14	101 ± 11	<0.001
RV (L)	3.63 ± 0.94	2.51 ± 0.52	<0.001
RV (% pred.)	153 (132–175)	108 (97–123)	<0.001
TLC (L)	6.97 ± 1.41	6.81 ± 1.23	0.635
TLC (% pred.)	105 ± 17	104 ± 13	0.953
**GOLD stage**			
GOLD 2 (%)	40 (87)	N/A	N/A
GOLD 3 (%)	6 (13)	N/A	N/A
**Comorbid conditions**			
Arterial hypertension, *n* (%)	30 (65.2)	14 (60.9)	0.723
Ischemic cardiac disease, *n* (%)	6 (13)	4 (17.4)	0.629
Dyslipidaemia, *n* (%)	22 (47.8)	12 (52.2)	0.733
Arrhythmias in the past, *n* (%)	4 (8.7)	2 (8.7)	1
**Beta-blocker use, ** ***n*** ** (%)**	14 (30.4)	7 (30.4)	1

* *p*-value < 0.05 indicates statistically significant results. Where applicable, values in the table are presented as the means ± SDs or medians (IQRs). Abbreviations: COPD—chronic obstructive pulmonary disease, BMI—body mass index, FEV_1_—forced expiratory volume in 1 s, FVC—forced vital capacity, RV—residual volume, TLC—total lung capacity, pred.—predicted, GOLD—Global Initiative for Chronic Obstructive Lung Disease, N/A—not applicable.

**Table 2:** Comparison of CPET measurements at peak exercise between COPD patients and non-COPD individuals.

Measurements	COPD (*n* = 46)	Non-COPD (*n* = 23)	*p*-Value *
**At rest**			
BORG-10 score	0 (0)	0 (0)	-
Leg pain score	0 (0)	0 (0)	-
HR, bpm	86 ± 12	80 ± 13	0.063
SpO_2_, %	96 (95–97)	97 (96–98)	0.007
**At peak exercise**			
RER	1.06 (0.96–1.08)	1.04 (1.01–1.08)	0.959
EET, s	456 ± 127	498 ± 119	0.191
BORG-10 score	5 (4–7)	4 (3–5)	0.018
Leg pain score	5 (4–7)	4 (3–5)	0.015
HR, bpm	130 ± 20	143 ± 17	0.012
HR, % pred.	82 ± 14	88 ± 9	0.025
SpO_2_, %	96 (92–97)	97 (96–98)	0.022
O_2_ pulse, mL/beat	11.5 ± 3.5	14.0 ± 2.4	0.003
O_2_ pulse, % pred.	73 ± 16	93 ± 18	<0.001
VO_2_, L/min	1.44 ± 0.43	1.97 ± 0.32	<0.001
VO_2_/kg, mL/kg/min	17.4 ± 4.4	22.8 ± 4.5	<0.001
VO_2_, % pred.	70.1 ± 16.5	98.7 ± 19.6	<0.001
ΔVO_2_/ΔWR	9.9 (7.8–10.7)	10.6 (9.4–12.2)	0.024
VE, L/min	49.3 ± 13.9	57.8 ± 12.0	0.015
VE, % pred.	73 (56–88)	77 (65–94)	0.09
VE/MVV	0.77 (0.68–0.91)	0.51 (0.39–0.70)	<0.001
VE/VCO_2_ slope	28.36 (25.87–32.73)	23.93 (21.84–26.12)	<0.001
WR, W	111 ± 42	152 ± 20	<0.001
**Reason for termination**			
Dyspnea	19	1	-
Leg pain	24	17	-
Hypertension	0	4	-
Ventricular extrasystoles	1	3	-
**Overall (for termination reason):**			<0.001

* *p*-value < 0.05 indicates statistically significant results. Where acceptable, the values in the table are presented as the means ± SDs or medians (IQRs), as appropriate. Abbreviations: COPD—chronic obstructive pulmonary disease, HR—heart rate, bpm—beats per minute, SpO_2_—peripheral oxygen saturation, RER—respiratory exchange ratio, EET—exercise endurance time, pred.—predicted, O_2_—oxygen (pulse), VO_2_—oxygen uptake, WR—work rate, VE—minute ventilation, MVV—maximal voluntary ventilation, VCO_2_—carbon dioxide output.

**Table 3:** Comparison of the SF-36 scores between COPD and non-COPD individuals.

SF-36 Domain	COPD (*n* = 46)	Non-COPD (*n* = 23)	*p*-Value *
Physical functioning	58 (49–71)	90 (80–95)	<0.001
RL due to physical health	25 (0–56)	100 (75–100)	<0.001
RL due to emotional problems	67 (25–100)	100 (67–100)	<0.001
Energy (fatigue)	53 ± 20	70 ± 14	<0.001
Emotional well-being	65 ± 18	79 ± 11	<0.001
Social functioning	78 (68–100)	90 (78–100)	0.025
Bodily pain	68 (47–93)	90 (78–100)	0.005
General health	44 ± 16	63 ± 12	<0.001
Health change	25 (0–50)	50 (50–50)	<0.001

* *p*-value < 0.05 indicates statistically significant results. The values in the table are presented as the means ± SDs or medians (IQRs), as appropriate. Abbreviations: COPD—chronic obstructive pulmonary disease, SF-36—the 36-item Short-Form Health Survey, RL—role limitations.

**Table 4:** Changes in the CPET parameters from baseline to 12 weeks of dual bronchodilation.

Measurements	Baseline (*n* = 32)	12 Weeks (*n* = 32)	*p*-Value *
**At rest**			
BORG-10 score	0 (0–1)	0 (0)	-
Leg pain score	0 (0–0)	0 (0–0)	-
HR, bpm	84 (77–89)	83 (76–89)	0.868
SpO_2_, %	96 (94–97)	97 (95–98)	0.073
**At peak exercise**			
RER	1.05 ± 0.12	1.04 ± 0.09	0.694
EET, s	472 ± 125	504 ± 112	0.162
BORG-10 score	5 (4–7)	5 (4–6)	0.750
Leg pain score	5 (4–7)	5 (4–7)	0.525
HR, bpm	130 ± 19	129 ± 13	0.808
HR, % pred.	81 ± 12	81 ± 9	0.913
SpO_2_, %	96 (93–97)	96 (94–97)	0.316
O_2_ pulse, mL/beat	11.3 (8.7–14.0)	12.5 (9.9–14.6)	0.003
O_2_ pulse, % pred.	74 ± 16	79 ± 16	0.015
VO_2_, L/min	1.52 (1.16–1.75)	1.59 (1.28–1.87)	0.019
VO_2_/kg, mL/kg/min	17.9 ± 4.2	18.9 ± 4.2	0.058
VO_2_, % pred.	70 ± 15	75 ± 16	0.044
ΔVO_2_/ΔWR	10.1 (9.0–10.7)	9.9 (9.1–10.5)	0.761
VE, L/min	51.62 ± 13.66	54.89 ± 14.51	0.128
VE, % pred.	74 (56–89)	76 (61–85)	0.074
VE/MVV	0.77 ± 0.23	0.69 ± 0.15	0.03
VE/VCO_2_ slope	27.7 (25.5–31.1)	28.1 (26.2–33.9)	0.076
WR, W	120 (90–140)	120 (100–139)	0.05

* *p*-value < 0.05 indicates statistically significant results. The values in the table are presented as the means ± SDs or medians (IQRs), as appropriate. Abbreviations: HR—heart rate, bpm—beats per minute, SpO_2_—peripheral oxygen saturation, RER—respiratory exchange ratio, EET—exercise endurance time, pred.—predicted, O_2_—oxygen (pulse), VO_2_—oxygen uptake, WR—work rate, VE—minute ventilation, MVV—maximal voluntary ventilation, VCO_2_—carbon dioxide output.

**Table 5:** Multivariable linear regression analysis of factors associated with the change in VE/MVV during 12 weeks of treatment with dual bronchodilation.

Predictor	B	95% CI	*p*-Value *
Baseline VE/MVV peak	0.71	0.46–0.96	<0.001
Beta-blocker use	−0.02	−0.13–0.09	0.716

* *p*-value < 0.05 indicates statistically significant results. Adjusted R^2^ = 0.541; overall model *p* < 0.001. Abbreviations: B—unstandardized regression coefficient, CI—confidence interval, VE/MVV—minute ventilation-to-maximum voluntary ventilation ratio.

**Table 6:** Changes in the SF-36 questionnaire scores from baseline to 12 weeks of dual bronchodilation.

SF-36 Domain	Baseline (*n* = 32)	12 Weeks (*n* = 32)	*p*-Value *
Physical functioning	58 ± 20	68 ± 20	0.002
RL due to physical health	25 (6–69)	25 (6–75)	0.546
RL due to emotional problems	67 (33–100)	33 (8–100)	0.330
Energy (fatigue)	52 ± 17	57 ± 19	0.152
Emotional well-being	65 ± 16	64 ± 17	0.827
Social functioning	70 (66–90)	78 (60–98)	0.280
Bodily pain	68 (50–88)	78 (45–90)	0.310
General health	44 ± 15	47 ± 14	0.109
Health change	25 (25–50)	50 (25–75)	<0.001

* *p*-value < 0.05 indicates statistically significant results. The values in the table are presented as the means ± SDs or medians (IQRs), as appropriate. Abbreviations: SF-36—the 36-item Short-Form Health Survey, RL—role limitations.

## Data Availability

The original contributions presented in the study are included in the article; further inquiries can be directed to the corresponding author.
